# Evaluation of Nutritional, Antioxidant, Antidiabetic, and Antidyslipidemic Properties of Red Corn Tortillas Enriched with *Moringa oleifera* Leaves

**DOI:** 10.3390/metabo16040252

**Published:** 2026-04-08

**Authors:** Eunice Tranquilino-Rodríguez, Noé Calderón-Téllez, José Juan Virgen-Ortiz, Juan de Dios Figueroa-Cárdenas, Rafael Zamora-Vega, José Octavio Rodiles-López, Héctor Eduardo Martínez-Flores

**Affiliations:** 1 Facultad de Químico Farmacobiología, Universidad Michoacana de San Nicolás de Hidalgo, Tzintzuntzan 173, Col. Matamoros, Morelia 58240, Mexico; eunice.tranquilino@umich.mx (E.T.-R.); 1414489j@umich.mx (N.C.-T.); rafael.zamora@umich.mx (R.Z.-V.); jose.rodiles@umich.mx (J.O.R.-L.); 2 SECIHTI–Centro de Investigacion en Alimentacion y Desarrollo, A. C. (CIAD)–CIDAM, Antigua Carretera a Patzcuaro Km 8, Morelia 58341, Mexico; jose.virgen@ciad.mx; 3 Centro de Investigación y de Estudios Avanzados del IPN, Unidad Querétaro, Libramiento Norponiente, No. 2000, Fracc. Real de Juriquilla, Santiago de Querétaro 76230, Mexico; jfigueroa@cinvestav.mx

**Keywords:** antioxidant activity, functional foods, metabolic disorders, *Moringa oleifera*, native corn, tortillas, polyphenols

## Abstract

**Highlights:**

**What are the main findings?**

- Tortillas enriched with *Moringa oleifera* significantly improved protein, fiber, mineral, and bioactive compound content.
- The enriched tortillas showed enhanced antioxidant activity and favorable effects on glucose and lipid metabolism in rats.

**What are the implications of the main findings?**

- Consumption of tortillas enriched with *Moringa oleifera* leaf was associated with lower serum triglycerides, total cholesterol, and hepatic lipid content, and a trend toward reduced glucose levels.
- Native corn and *Moringa oleifera* can be valorized as sustainable ingredients for health-promoting foods.

**Abstract:**

**Background/Objectives:** Metabolic diseases are increasingly associated with diets low in bioactive compounds. Native maize varieties possess functional potential; however, they remain underutilized. *Moringa oleifera* leaf flour (MF), rich in protein and polyphenols, represents a promising functional ingredient. This study evaluated the incorporation of MF into red native corn tortillas and its effects on nutritional composition and antioxidant capacity, as well as assessed its hypoglycemic and hypolipidemic effects in Wistar rats. **Methods:** Tortillas were formulated with 5% MF. Nutritional composition was determined using standard AOAC methods, while bioactive compounds (total phenolics and flavonoids) and antioxidant activity were evaluated using Folin–Ciocalteu, aluminum chloride (AlCl_3_) colorimetric, DPPH^•^, and ABTS^•+^ assays, respectively. Male Wistar rats (12 weeks old, with an approximate weight ofs 360 g; *n* = 5/group) were fed the experimental diets for 21 days with either a standard diet, a high-fat diet, or high-fat diets supplemented with MF or MF-enriched tortillas. Serum glucose, triglycerides, total cholesterol, and HDL were measured using enzymatic colorimetric methods. Data were analyzed by ANOVA followed by Tukey’s test (*p* < 0.05). **Results:** MF incorporation increased protein (+19.85%), dietary fiber (+18.51%), and mineral content (+41.03%) compared to control tortillas. Total phenolics and flavonoids increased by 114.0% and 184.7%, respectively. Antioxidant activity improved significantly, as evidenced by reductions in IC_50_ values of 41.1% (DPPH^•^) and 43.1% (ABTS^•^). In vivo, MF-enriched tortillas reduced triglycerides by 68.4%, total cholesterol by 16.2%, and hepatic lipid accumulation by 31.8% compared to the high-fat diet group. Glucose levels showed a reduction of 8.5%, although not statistically significant (*p* > 0.05). **Conclusions:** The incorporation of MF into red corn tortillas significantly enhances their nutritional and functional properties. In vivo results also showed improvements in lipid profile and a non-significant reduction in glucose levels. These findings support the development of functional foods based on traditional staples with potential health benefits.

## 1. Introduction

The global increase in non-communicable chronic diseases, particularly obesity and diabetes mellitus, has driven growing interest in functional ingredients capable of promoting health. The International Diabetes Federation estimates that the number of people living with diabetes will reach 643 million by 2030 and 783 million by 2045. In addition, it is projected that by 2030, approximately 20% of women and 14% of men worldwide will present clinical obesity [1,2]. In this context, the development of accessible and culturally accepted functional foods represents a promising strategy for the prevention and management of these conditions.

Maize is one of the most important crops worldwide, with a production exceeding 1.2 billion tons [3]. Pigmented varieties have attracted increasing attention due to their distinctive phytochemical profile, particularly their high content of polyphenols such as anthocyanins and flavonoids, which are associated with elevated antioxidant capacity and potential nutraceutical properties [4,5]. In Mexico, maize is primarily consumed in the form of tortillas. The traditional nixtamalization process, which involves high temperatures and alkaline conditions, modifies the cell wall matrix and releases previously bound phenolic compounds, thereby increasing their bioaccessibility and bioavailability [6]. Consequently, the regular consumption of maize-based products, especially those derived from pigmented varieties, may contribute to reducing the risk of non-communicable chronic diseases due to their antioxidant content [7].

*Moringa oleifera* is a deciduous tree of the Moringaceae family, widely cultivated in Asia, Africa, and other tropical regions due to its rapid growth and tolerance to drought and nutrient-poor soils [8]. Its leaves represent the most nutritionally valuable part of the plant, containing significant amounts of minerals (calcium, iron, potassium, and phosphorus), vitamins (A, B-complex, and C), unsaturated fatty acids, high-quality protein with all essential amino acids, and dietary fiber [9]. In addition to its nutritional value, *M. oleifera* is recognized for its high content of bioactive compounds, particularly polyphenols such as quercetin, kaempferol, and rutin, which have been associated with hypoglycemic, lipid-lowering, and antihypertensive effects [10]. These flavonoids have been reported to reduce intestinal cholesterol absorption, improve lipid metabolism, and attenuate liver alterations induced by high-fat diets [11,12]. Furthermore, the synergistic action of phenolic compounds may promote insulin secretion and enhance peripheral glucose utilization through the inhibition of hepatic gluconeogenesis [13].

The incorporation of *M. oleifera* into food products has been shown to improve their nutritional quality and phytochemical content without significantly affecting sensory properties, as demonstrated in bread, dairy products, soups, and beverages [14]. Therefore, supplementation with *M. oleifera* in commonly consumed foods such as corn tortillas may enhance the intake of bioactive compounds with beneficial health effects. The aim of the present study was to evaluate the incorporation of *M. oleifera* leaves into tortillas made from red pigmented maize and to determine its effects on nutritional composition, antioxidant capacity, and hypoglycemic and hypolipidemic parameters in Wistar rats.

## 2. Materials and Methods

### 2.1. Biological Samples

Red native maize variety was collected in Arantepacua, a municipality of Nahuatzen, Michoacán, Mexico. The taxonomic identification and classification of the plant material were carried out by the Herbarium of the Faculty of Biology (EBUM) at the Universidad Michoacana de San Nicolás de Hidalgo, where was identified as *Zea mays* L. subsp. *Mays* and was registered under Voucher No. 18624. *Moringa oleifera* leaves were collected in Salguero, Michoacán, Mexico. Their taxonomic identification was performed by the same herbarium and assigned Voucher No. 18610.

### 2.2. Preparation of Moringa oleifera Leaf Flour

*M. oleifera* leaves were disinfected by immersion in 70% ethanol for 5 min. Subsequently, the leaves were air-dried in the shade at room temperature for 72 h. The dried material was ground and sieved to obtain flour with a particle smaller than 250 μm. The resulting *M. oleifera* flour (MF), was stored at 4 °C until further analysis.

### 2.3. Production of Nixtamalized Corn Flours

The process for producing tortillas through nixtamalization followed the procedure described by Zavala [15]. One kilogram of corn kernels was combined with 2 L of potable water and 10 g of calcium hydroxide, and the mixture was heated on a gas stove to 92 °C for 40 min. After cooking, the kernels were allowed to steep in the same cooking liquid (nejayote) for 16 h at room temperature. Subsequently, the nejayote was decanted, and the nixtamalized corn kernels were washed with 2 L of potable water per kilogram of corn to remove excess lime and part of the detached pericarp. The resulting nixtamal was ground using a stone mill (M100, FUMASA, Puebla, Mexico) to obtain masa, which was then dehydrated using a flash-type drying system and pulverized until flour with a homogeneous particle size was obtained.

### 2.4. Preparation of Corn Tortillas and Tortillas Fortified with Moringa oleifera Leaf

Two experimental treatments were formulated: red corn tortilla (CT) with 0.5% carboxymethylcellulose, and red corn tortilla with 0.5% carboxymethylcellulose and supplemented with 5% *M. oleifera* leaf flour (CTMF5%). The nixtamalized corn flours were rehydrated with potable water until reaching a moisture content of 60% to obtain homogeneous doughs. Portions of 30 g were formed and pressed using a tortilla press machine to produce discs approximately 13.5 cm in diameter and 1.2 mm thick. Cooking was carried out on an iron griddle preheated to 250 °C. Each tortilla was cooked for 17 s on the first side, 55 s on the second side, and an additional 17 s on the first side.

### 2.5. Chemical Composition of Tortillas

The nutritional composition of CT, MF and CTMF5% were determined using official AOAC International methods [16]. Ash content was quantified by incineration in a muffle furnace (AOAC 923.03). Total protein content was determined using the Kjeldahl method (AOAC 979.09). Crude fat was quantified by solvent extraction using the Soxhlet method (AOAC 920.39). Total, soluble, and insoluble dietary fiber were determined using the enzymatic–gravimetric method (AOAC 985.29). The carbohydrate content was estimated by difference according to the Equation (1):

Carbohydrates (%) = 100 − (% moisture + % ashes + % fat + % protein + % dietary fiber)(1)

### 2.6. Amino Acid Quantification

Samples were subjected to acid hydrolysis to release amino acids; during this process, asparagine and glutamine were converted into aspartic acid and glutamic acid, respectively. Tryptophan was determined by a separate alkaline hydrolysis. Amino acids were quantified by high-performance liquid chromatography (HPLC) using an Agilent 1200 Infinity Series system (Agilent Technologies, Santa Clara, CA, USA). Prior to analysis, pre-column derivatization was performed with o-phthalaldehyde for primary amino acids and with 9-fluorenylmethyl chloroformate for secondary amino acids, following the protocol described by Henderson et al. [17].

### 2.7. Evaluation of Tortilla Quality Parameters

Various physical and mechanical properties were evaluated with five repetitions, including puffing degree, cooking weight loss, tensile strength, cutting force, and rollability.

Puffing degree: Tortillas were cooked and the percentage of puffed surface area was visually estimated using a scale from 1 to 3 (1 = 75–100%; 2 = 25–75%; 3 = 0–25%). Breakage degree: A scale from 1 to 5 was used, where 1 indicated no rupture and 5 indicated complete rupture.

Cooking weight loss: The cooking conditions for the tortillas are detailed in Section 2.4, where the tortilla preparation process is described. For the determination of cooking weight loss, this parameter was calculated as the difference between the weight of the tortilla before and after cooking. Each tortilla was cooked under the conditions described in Section 2.4, and the weight loss was expressed as a percentage of the initial weight.

For tensile strength a piece of tortilla measuring 32 length × 16 length was fixed in retention pincers TA-26 of the Brookfield CT3 Texture Analyzer equipped (Brookfield Engineering Laboratories, Middleboro, MA, USA). The test was carried out at 2 mm/s and pincels were opened 10 mm until the tortilla piece was broken, and the maximum force (N) was recorded.

To measure the cutting force, a piece of tortilla was cut using a flat blade in the central area of the tortilla at a speed of 2 mm/s and a cutting depth of 10 mm, recording the force required for cutting (N).

Rollability was evaluated by rolling a tortilla over a 2 cm diameter tube. The extent of breaking was quantified, using a scale of 1 to 5, where 1 corresponded to tortillas with no breaking; 2, breaking of the outer edges of tortilla; 3, partial breaking at the center and edges of the tortillas; 4, breaking of several parts; and 5, those that were completely flattened.

### 2.8. Extraction and Determination of Bioactive Compounds and Antioxidant Activity

The extraction of phenolic compounds was performed using 30% ethanol at a 1:20 (*w*/*v*) ratio, assisted by ultrasound equipment (VC505; Sonics & Materials, Inc., Newtown, CT, USA) for 20 min at 50 °C, with an amplitude of 30% and cycles of 30 s of sonication followed by 30 s of rest. The extracts were centrifuged at 3000 rpm for 15 min and subsequently vacuum-filtered using Whatman No. 1 filter paper. From the extract, determinations of total phenolic compounds, total flavonoid compounds, and antioxidant capacity through DPPH^•^ and ABTS^•+^ radical scavenging assays were performed.

Total phenolic content was quantified using the Folin–Ciocalteu method described by Makkar et al. [18], with minor modifications proposed by Treviño-Gómez et al. [19], using gallic acid as the standard and measuring absorbance at 750 nm. The results were expressed as mg GAE/g of dry sample.

Total flavonoids were determined using the AlCl_3_ colorimetric method reported by Liu et al. [20], employing quercetin as the standard and measuring absorbance at 510 nm. The results were expressed as mg QE/g of dry sample.

Anthocyanins were quantified using the differential pH method described by Rapisarda et al. [21]. Water was used as the extraction solvent, and absorbance was measured at 510 nm in buffer solutions at pH 1.0 and 4.5 [22,23]. Anthocyanin concentration was calculated using the molecular weight and molar absorptivity of cyanidin-3-glucoside, along with the appropriate dilution factor, following the standard equation for this method. Results were expressed as µg of cyanidin-3-glucoside equivalents (C3G) per 100 g of dry sample.

Antioxidant activity was evaluated using 2,2-diphenyl-1-picrylhydrazyl (DPPH^•^) and 2,20-azino-bis(3-ethylbenzothiazoline-6-sulfonic acid) (ABTS^•+^) radical scavenging assays, following the methods described by Randhir and Shetty [24] and Re et al. [25], respectively. In both cases, antioxidant capacity was expressed as IC_50_ (mg/mL). Absorbance readings were taken at 517 nm (DPPH^•^) and 734 nm (ABTS^•+^).

### 2.9. Biological Assay

#### 2.9.1. Experimental Animals and Diets

Twenty-five male Wistar rats (12 weeks old, average weight 360 g) were used. The sample size (n = 5 per group) was determined based on literature studies with similar experimental conditions and a power analysis, which indicated that this number would be sufficient to detect a minimum effect size with a significance level of 0.05. Animal handling and care followed the regulations established by the Official Mexican Standard NOM-062-ZOO-1999 [26]. This study was approved by the Ethics Committee in Research and Biosafety of the Michoacan University of San Nicolás de Hidalgo on 22 January 2026 (approval protocol code: CEIIB-UMSNH-015-2026). All procedures were designed to minimize animal suffering and reduce the number of animals used. Animals were housed individually at 22 °C under 12-h light–dark cycles, with ad libitum access to food and water. After a 7-day acclimation period, animals were randomly assigned to five groups (n = 5) using a computer-generated randomization schedule. Animals were fed for 21 days with the following diets: Group 1: Fed with control diet (CD), based on standard AIN-93 diet (382 kcal/100 g). Group 2: Fed with high-fat diet (HFD) (428 kcal/100 g). Group 3: Fed with high-fat diet with 2.5% *M. oleifera* flour (HFD-M) (425 kcal/100 g). Group 4: Fed with high-fat diet with 59% CT (HFD-T) (428 kcal/100 g). Group 5: Fed with high-fat diet with *M. oleifera* flour (HFD-T-M) (428 kcal/100 g). The diets were formulated according to the recommendations of Reeves et al. [27], with ingredient composition adjusted to meet the specific requirements of each diet. The following considerations were taken into account in the formulation of the experimental diets: (1) Diets HFD, HFD-M, HFD-T, and HFD-T-M (groups 2–5) were designed to be as isocaloric and isoproteic as possible, with an energy range of 425–428 kcal/100 g. To achieve this, the proportions of starch, casein, and soybean oil were adjusted, particularly in diets HFD-M, HFD-T, and HFD-T-M (groups 3–5), which included *M. oleifera* flour, red corn tortillas, and red corn tortillas enriched with *M. oleifera* flour, respectively. The macronutrient contribution (carbohydrates, protein, and lipids) of each ingredient was considered during formulation to ensure nutritional consistency among diets. (2) Red corn tortillas were formulated with 5% *M. oleifera* flour. When incorporated into diet 5 at a 50% inclusion level, the final diet contained 2.5% *M. oleifera* and 47.5% red corn. Based on this, diet HFD-M (group 3) was formulated to contain 2.5% *M. oleifera* flour alone, to isolate and evaluate its specific effects on serum triglycerides, cholesterol fractions, and glucose levels in the Wistar rat model. This approach allowed for differentiation between the effects of *M. oleifera* alone and its combined effect within the tortilla matrix.

The composition of the diets is detailed in Table 1. Food intake and body weight were recorded daily. At the end of the experiment, the animals were anesthetized with sodium pentobarbital (50 mg/kg, intraperitoneally) and subsequently euthanized by exsanguination via cardiac puncture. Blood samples were collected, and liver tissues were excised, weighed, and subjected to further analysis.

#### 2.9.2. Serum Analyses

Serum samples were analyzed for concentrations of glucose, triglycerides, total cholesterol, and HDL-cholesterol using colorimetric enzymatic methods with commercial in vitro diagnostic (IVD) reagents from Spinreact (Girona, Spain). Glucose was determined by the glucose oxidase–peroxidase (GOD-POD) method based on the Trinder reaction, using a commercial IVD kit. Triglycerides were measured using the glycerol-3-phosphate oxidase–peroxidase (GPO-POD) colorimetric enzymatic method with a commercial IVD reagent. Total cholesterol was quantified using the cholesterol oxidase–peroxidase (CHOD-POD) method with the Cholesterol-LQ kit. HDL-cholesterol (HDL-C) was determined after selective precipitation of non-HDL lipoproteins using a commercial precipitating reagent (HDL-cholesterol, precipitation method, Spinreact), followed by enzymatic quantification of cholesterol in the supernatant using the CHOD-POD method. All measurements were performed on a Mindray BS-240 Vet automated chemistry analyzer (Shenzhen, China).

#### 2.9.3. Moisture and Total Lipids in Liver

Three grams of liver per animal were dehydrated in an oven at 105 °C for 3 h to determine moisture content [19]. Subsequently, the dried samples were subjected to Soxhlet extraction for the quantification of total lipids [19].

### 2.10. Statistical Analysis

Results are expressed as mean ± standard deviation (n = 5). Data normality was assessed using the Shapiro–Wilk test prior to statistical analysis. Differences among groups were analyzed using one-way analysis of variance (ANOVA), followed by Tukey’s post hoc test. The significance level of *p* < 0.05 was used to determine statistically significant differences. All statistical analyses were performed using JMP (version 15; SAS Institute, Cary, NC, USA).

## 3. Results

### 3.1. Nutritional Composition, Phenolic Compounds, and Antioxidant Activity

#### 3.1.1. Proximate Chemical Analysis

Table 2 presents the proximate chemical composition of *M. oleifera* leaf flour (MF), red corn tortillas (CT), and tortillas supplemented with 5% MF (CTMF5%). Due to the high protein content of MF (31.84%), CTMF5% showed a protein content of 9.54%, increasing to 19.84% compared to CT. Dietary fiber and mineral content also increased significantly (*p* < 0.05) from 8.43% to 9.99% and from 1.56% to 2.20%, respectively. In contrast, carbohydrate content decreased from 79.89% to 76.10%.

#### 3.1.2. Amino Acid Content

The amino acid profiles (Table 3) showed that CTMF5% increased nearly all essential amino acids compared to CT. Total essential amino acids increased from 385.0 to 396.7 g/kg protein (2.94%). Lysine content increased by 23.22%, while leucine reaches a value of 115.64 mg/g of protein.

#### 3.1.3. Phenolic Compounds and Antioxidant Activity

As shown in Table 4, supplementation with MF significantly increased (*p* < 0.05) phenolic compounds and antioxidant activity. CTMF5% showed increases of 114.03% in total phenolics and 184.74% in flavonoids. Antioxidant activity increased by 69.83% (DPPH^●^) and 75.73% (ABTS^●+^). Total anthocyanin content was 3.53 µg C3G/100 g in *M. oleifera* flour, 149.99 mg/100 g in corn tortillas (CT), and 148.02 µg C3G/100 g in corn tortillas supplemented with *M. oleifera* flour (CTMF5%). No significant differences were observed between CT and CTMF5% (*p* > 0.05), indicating that the addition of moringa did not alter the anthocyanin content of the tortillas.

### 3.2. Quality Parameters of Tortillas

As shown in Table 5, puffing degree and weight loss did not differ significantly between CT and CTMF5% (*p* > 0.05), with puffing degree averaging 1.67.

In texture analysis, CTMF5% exhibited higher cutting shear force (31.8 N) compared to CT (1.80 N). Tensile strength showed no significant differences (*p* > 0.05), although a slight increase was observed in CTMF5% (3.82 N).

### 3.3. Biological Assay

#### 3.3.1. Hypoglycemic and Hypolipidemic Effects in Rodents

Initial body weights showed no significant differences among groups (Table 6). All high-fat diet groups exhibited increased weight gain, with the highest in G4 (CT) and the lowest in G3 (MF). No differences in feed intake were observed.

As shown in Table 7, HFD (high-fat diet) presented significantly higher glucose levels (*p* < 0.05) compared to CD. Groups HFD-M, HFD-T and HFD-T-M showed a reduction trend, with decreases of 15.21%, 10.72%, and 8.51%, respectively.

Triglyceride levels showed a decreasing trend in supplemented groups, with reductions of 68.36% (HFD-T-M), 48.67% (HFD-M), and 44.30% (HFD-T) relative to HFD.

Total cholesterol increased significantly in HFD (*p* < 0.05). Supplemented groups showed reductions of 21.00% (HFD-M), 19.01% (HFD-T), and 16.17% (HFD-T-M). HDL levels decreased in HFD; however, they increased in HFD-T-M (46.90 mg/dL), approaching control values.

#### 3.3.2. Liver Parameters

Table 8 shows a significant increase (*p* < 0.05) in liver weight in all high-fat diet groups. Moisture content decreased in HFD, however, increased in supplemented groups. Hepatic lipid content was 6.3 times higher in HFD than in CD.

Supplemented diets significantly reduced liver lipids (*p* < 0.05), with reductions of 31.78% (HFD-T-M), 23.03% (HFD-T), and 22.31% (HFD-M).

## 4. Discussion

The improvement in protein, dietary fiber, and mineral content observed in CTMF5% is directly attributable to the high nutritional density of *M. oleifera* flour. Beyond the quantitative increase, the enhancement in essential amino acid composition, particularly lysine, has important nutritional implications, as lysine is typically the limiting amino acid in cereal-based foods. This suggests that the formulation not only increases protein content but also improves protein quality, which is a critical factor in plant-based diets [28]. In addition, the increase in dietary fiber may have relevant physiological implications, as dietary fiber is known to promote satiety and reduce overall energy intake, which is a key factor in the prevention and management of obesity and related metabolic disorders [29]. This suggests that the formulation not only improves nutrient composition but may also contribute to appetite regulation.

The marked increase in total phenolics, flavonoids, and antioxidant activity confirms that *M. oleifera* acts as a potent source of bioactive compounds when incorporated into a food matrix, such as tortillas. The magnitude of increase (>100% in total phenolic compounds and flavonoid total compounds) indicates that even low inclusion levels (5%) are sufficient to significantly enhance functional properties, consistent with previous reports in moringa-enriched foods [30]. In contrast, the reduced anthocyanin content in control tortillas highlights the impact of nixtamalization on thermolabile compounds, as previously reported by Robledo-Márquez et al. [31], suggesting that processing conditions remain a critical factor influencing the final bioactive profile.

From a functional perspective, the combination of red corn and *M. oleifera* represents a relevant strategy for designing foods with complementary bioactive profiles. Anthocyanins from pigmented corn and flavonoids from *M. oleifera* may exert synergistic effects through overlapping mechanisms, including reactive oxygen species scavenging and modulation of signaling pathways related to oxidative stress and inflammation [32,33,34,35]. This synergy may partially explain the enhanced antioxidant capacity observed in CTMF5%.

The technological evaluation showed that the incorporation of *M. oleifera* slightly modified textural properties, likely due to its high fiber content and its effect on water absorption and starch matrix interactions. Although most parameters were not significantly affected, a significant increase in cutting force was observed in the supplemented tortillas. However, these changes did not compromise critical quality attributes such as puffing degree, breakage, or tensile strength, indicating that the formulation is technologically viable. Similar observations have been reported in tortillas supplemented with *M. oleifera* flour [36].

In the in vivo model, the tendency to reduce glucose levels observed in the supplemented groups may be associated with the presence of bioactive compounds known to influence glucose metabolism. Flavonoids and related compounds in *M. oleifera* have been associated with improved insulin signaling and glucose uptake [37,38]; however, these mechanisms were not directly evaluated in the present study. Additionally, anthocyanins present in red corn have been associated with improved insulin sensitivity and antioxidant effects in previous studies [39,40]. Nevertheless, because no direct measurements of oxidative stress markers, insulin signaling, or specific glucose transport mechanisms were performed, further studies are required to corroborate these potential effects and clarify the underlying mechanisms.

The lipid-lowering effects observed, particularly the pronounced reduction in triglycerides in the HFD-T-M group, further supports a potential synergistic interaction between the bioactive compounds of both ingredients. Phenolic compounds such as quercetin and kaempferol as well as saponins have been reported to modulate lipid metabolism by inhibiting lipogenesis and promoting fatty acid oxidation [11,41,42]. The presence of these compounds in *M. oleifera* leaf flour, previously identified by our research group [43], may explain the improvements observed in the lipid profile.

The reduction in hepatic lipid accumulation in supplemented groups is particularly relevant, as it suggests a protective effect against diet-induced steatosis. This effect may be mediated by the regulation of pathways such as AMPK activation and reduced expression of lipogenic genes, mechanisms previously associated with both moringa-derived compounds and anthocyanins [34,44]. The stronger response observed in HFD-T-M supports the hypothesis of additive or synergistic interactions at the hepatic level.

Most previous studies have evaluated *M. oleifera* in extract form, which limits their applicability to real dietary conditions. In this context, the present study provides evidence that its incorporation into a traditional food matrix preserves its biological activity and exerts measurable metabolic effects. Similar findings have been reported in food-based applications, such as *M. oleifera*-supplemented products with reduced glycemic and lipid responses [45].

The high-fat diet (HFD) model used in this study was designed to induce early metabolic alterations, including mild hyperglycemia, dyslipidemia, and hepatic lipid accumulation, which are commonly associated with the initial stages of metabolic syndrome. This approach allows the evaluation of preventive effects of dietary interventions rather than therapeutic effects in advanced disease states.

Overall, this study highlights the potential of combining underutilized native crops with bioactive-rich plant ingredients to develop functional foods with metabolic benefits. Such strategies may contribute to the prevention of hyperglycemia and dyslipidemia, as well as to the promotion of sustainable food systems and the revalorization of traditional dietary components.

## 5. Conclusions

This study demonstrates that incorporating *Moringa oleifera* leaves into red corn tortillas markedly enhances their nutritional quality, including increased lysine, dietary fiber, and mineral content, as well as their bioactive potential, evidenced by a twofold increase in phenols and flavonoids and corresponding improvements in antioxidant activity. Additionally, over the 3-week intervention, the biological model revealed a synergistic effect of the two components in the red corn tortilla with *M. oleifera* on lipid metabolism, resulting in significant reductions in serum triglycerides (68.4%) and liver lipid content (31.8%) in rats fed high-fat diets. Diet based on corn tortilla with added *M. oleifera* also showed beneficial trends in glucose homeostasis. These findings suggest a potential role in the early modulation of glucose and lipids, although further studies with larger sample sizes are needed to confirm these effects.

## Figures and Tables

**Table 1:** Ingredients for the formulation of different diets.

Ingredient (%)	CD	HFD	HFD-M	HFD-T	HFD-T-M
*M. oleifera* flour	-	-	2.5	-	-
Red corn tortillas	-	-	-	59	-
Red corn tortillas + *M. oleifera* flour	-	-	-	-	50 (47.5% corn tortillas and 2.5% *M. oleifera* flour)
Starch	71.5	60.5	60.0	13.93	23.0
Soybean oil	4.0	4.0	3.86	2.77	2.90
Casein	15.0	15.0	14.27	9.7	9.7
Mineral mix	3.5	3.5	3.27	2.6	2.4
Vitamin mix	1.0	1.0	1.0	1.0	1.0
Cellulose	5.0	5.0	4.1	0.0	0.0
Coconut oil	-	10.0	10	10.0	10.0
Cholesterol	-	1.0	1.0	1.0	1.0
Total	100	100	100	100	100
kcal/100 g	382	428	425	428	428

CD: Control diet, based on standard AIN-93 diet. HFD: High-fat diet. HFD-M: High-fat diet with 2.5% *M. oleifera* flour. HFD-T: High-fat diet added with 59% corn tortilla. HFD-T-M: High-fat diet added with corn tortilla and *Moringa oleifera* flour.

**Table 2:** Proximate chemical analysis of processed foods.

Chemical Component (%)	MF	CT	CTMF5%
Protein	31.84 ± 1.07 ^a^	7.96 ± 1.20 ^b^	9.54 ± 1.10 ^b^
Fat	5.88 ± 0.40 ^a^	2.09 ± 0.30 ^b^	2.20 ± 0.04 ^b^
Dietary fiber	41.30 ± 0.75 ^a^	8.43 ± 0.50 ^c^	9.99 ± 0.70 ^b^
of which:			
Soluble fiber	6.37 ± 0.95 ^a^	1.04 ± 0.30 ^b^	1.29 ± 0.20 ^b^
Insoluble fiber	34.52 ± 2.02 ^a^	7.39 ± 0.50 ^c^	8.70 ± 0.70 ^b^
Ashes	9.72 ± 0.05 ^a^	1.56 ± 0.20 ^b^	2.20 ± 0.60 ^c^
Carbohydrates	11.24 ± 0.66 ^a^	79.89 ± 1.2 ^b^	76.10 ± 0.90 ^c^

The data presented are the mean of the measurements ± standard deviation. N = 3. Different letters in the same row indicate statistically significant differences with a significance level of *p* < 0.05. (ANOVA, Tukey’s test). MF = *M. oleifera* flour. CT = Red corn tortilla. CTMF5% = Red corn tortilla + *M. oleifera* flour 5%.

**Table 3:** Amino acid content in the processed products.

Amino Acids	MF	CT	CTMF5%	FAO and IAEA (2024) (mg/g of Protein)
Histidine	18.18	21.08	23.68	15
Isoleucine	50.29	32.58	34.66	30
Leucine	10.44	120.11	115.64	59
Lysine	102.13	27.47	33.85	45
Methionine + cystine	26.31	27.47	25.99	22
Phenylalanine + tyrosine	78.53	64.66	67.24	38
Threonine	54.16	35.01	37.08	23
Tryptophan	11.61	7.28	8.90	6
Valine	63.83	49.32	49.68	30
Alanine	59.19	73.47	71.05	-
Arginine	70.79	38.46	41.47	-
Aspartic acid	112.57	62.87	67.12	-
Glycine	50.68	37.57	37.55	-
Glutamic acid	229.01	208.41	201.25	-
Serine	41.01	48.94	48.52	-
Proline	21.28	85.61	77.98	-

MF = *M. oleifera* flour. CT = Red corn tortilla. CTMF5% = Red corn tortilla + *M. oleifera* flour 5%.

**Table 4:** Phenolic compound content and antioxidant activity in the processed products.

Parameter	MF	CT	CTMF5%
Total phenolics (mg GAE/100 g)	1213.46 ± 2.70 ^a^	39.39 ± 6.90 ^c^	84.31 ± 9.02 ^b^
Total flavonoids (mg QE/100 g)	767.43 ± 1.80 ^a^	12.52 ± 1.30 ^c^	35.65 ± 1.80 ^b^
Anthocyanins (µg C3G/100 g)	3.53 ± 0.80 ^b^	149.99 ± 2.20 ^a^	148.02 ± 0.50 ^a^
IC_50_ DPPH^●^ (μg/mL)	160.29 ± 2.20 ^c^	822.32 ± 10.40 ^a^	484.19 ± 5.50 ^b^
IC_50_ ABTS^●+^ (μg/mL)	42.78 ± 4.80 ^c^	651.05 ± 2.80 ^a^	370.48 ± 4.80 ^b^

The data presented are the mean of the measurements ± standard deviation. N = 3. Different letters in the same row indicate statistically significant differences with a significance level of *p* < 0.05. (ANOVA, Tukey’s test). MF = *M. oleifera* flour. CT = Red corn tortilla. CTMF5% = Red corn tortilla + *M. oleifera* flour 5%.

**Table 5:** Quality parameters of the tortillas produced.

Parameter	CT	CTMF5%
Puffing grade	1.67 ± 0.58 ^a^	1.67 ± 0.58 ^a^
Rollability	1.0 ± 0.0 ^a^	2.0 ± 1.0 ^a^
Weight loss (%)	4.83 ± 0.71 ^a^	5.27 ± 0.60 ^a^
Cutting shear force (N)	1.80 ± 0.08 ^b^	3.18 ± 0.20 ^a^
Tensile strength (N)	2.38 ± 0.01 ^a^	3.82 ± 0.17 ^ab^

The data presented are the mean of the measurements ± standard deviation. N = 3. Different letters in the same row indicate statistically significant differences with a significance level of *p* < 0.05. (Student *t*-test). CT = Red corn tortilla. CTMF5% = Red corn tortilla + *M. oleifera* flour 5%.

**Table 6:** Initial weight, weight gain, and food consumed by the animals in experimentation.

Group	Initial Weight (g)	Weight Gain (g)	Daily Feed Intake (g)
CD	352.66 ± 12.02 ^a^	49.42 ± 8.15 ^a^	26.09 ± 1.29 ^a^
HFD	358.52 ± 24.44 ^a^	68.12 ± 19.47 ^a^	25.62 ± 2.27 ^a^
HFD-M	357.44 ± 23.33 ^a^	55.36 ± 20.48 ^a^	23.21 ± 2.56 ^a^
HFD-T	365.36 ± 26.37 ^a^	75.32 ± 18.90 ^a^	24.11 ± 2.45 ^a^
HFD-T-M	363.30 ± 18.45 ^a^	62.08 ± 23.49 ^a^	24.32 ± 2.87 ^a^

CD: Control diet. HFD: High-fat diet. HFD-M: High-fat diet with *M. oleifera* flour. HFD-T: High-fat diet added with red corn tortilla. HFD-T-M: High-fat diet added with red corn tortilla + *M. oleifera* flour. The data presented are the mean of measurements standard deviation. N = 5. Different letters in the same column indicate statistically significant differences with a significance level of *p* < 0.05. (ANOVA, Tukey’s test).

**Table 7:** Serum parameters of the animals in the experiment.

Group	Glucose (mg/dL)	Triglycerides (mg/dL)	Total Cholesterol (mg/dL)	HDL-Cholesterol (mg/dL)
CD	133.72 ± 4.99 ^b^	63.69 ± 34.44 ^ab^	56.04 ± 11.76 ^b^	47.40 ± 10.71 ^a^
HFD	167.76 ± 23.03 ^a^	80.98 ± 42.20 ^a^	77.30 ± 17.91 ^a^	30.40 ± 4.33 ^a^
HFD-M	142.24 ± 14.31 ^ab^	41.56 ± 10.56 ^ab^	61.06 ± 5.03a ^b^	24.20 ± 3.42 ^a^
HFD-T	149.76 ± 11.86 ^ab^	45.10 ± 12.03 ^ab^	62.60 ± 5.74a ^b^	30.20 ± 4.65 ^a^
HFD-T-M	153.48 ± 15.50 ^ab^	25.62 ± 12.87 ^b^	64.80 ± 6.88a ^b^	46.90 ± 26.48 ^a^

CD: Control diet. HFD: High-fat diet. HFD-M: High-fat diet added with *M. oleifera* flour. HFD-T: High-fat diet added with red corn tortilla. HFD-T-M: High-fat diet added with red corn tortilla + *M. oleifera* flour. The data presented are the mean of the measurements ± standard deviation. N = 5. Different letters in the same column indicate statistically significant differences with a significance level of *p* < 0.05. (ANOVA, Tukey’s test).

**Table 8:** Weight, moisture and lipid parameters in liver.

Group	Liver Weight (g)	Liver Moisture (%)	Liver Lipids (%)
CD	12.90 ± 1.45 ^a^	70.15 ± 0.43 ^a^	3.75 ± 0.15 ^d^
HFD	17.48 ± 1.96 ^b^	54.46 ± 1.54 ^c^	23.75 ± 0.66 ^a^
HFD-M	17.21 ± 1.95 ^b^	59.07 ± 1.49 ^b^	18.45 ± 0.25 ^b^
HFD-T	17.75 ± 1.09 ^b^	59.19 ± 0.83 ^b^	18.28 ± 0.11 ^b^
HFD-T-M	17.52 ± 0.61 ^b^	60.22 ± 1.47 ^b^	16.20 ± 0.42 ^c^

CD: Control diet. HFD: High-fat diet. HFD-M: High-fat diet added with *M. oleifera* flour. HFD-T: High-fat diet added with red corn tortilla. HFD-T-M: High-fat diet added with red corn tortilla + *M. oleifera* flour. The data presented are the mean of measurements ± standard deviation. N = 5. Different letters in the same column indicate statistically significant differences with a significance level of *p* < 0.05 (ANOVA, Tukey’s test).

## Data Availability

The data of this article will be made available by the authors on request.

## Abbreviations

The following abbreviations are used in this manuscript:

MF	*Moringa oleifera* flour
CT	Red corn tortilla
CTMF5%	Red corn tortilla supplemented with 5% *Moringa oleifera* leaf flour
HPLC	High-performance liquid chromatography
DPPH^•^	2,2-diphenyl-1-picrylhydrazyl
ABTS^•+^	2,20-azino-bis(3-ethylbenzothiazoline-6-sulfonic acid)
ANOVA	Analysis of variance
CD	Control diet
HFD	High fat diet
HFD-M	High fat diet added with *Moringa oleifera*
HFD-T	High fat diet added with red corn tortilla
HFD-T-M	High fat diet added with *Moringa oleifera* flour
